# A Metabotropic-Like Flux-Independent NMDA Receptor Regulates Ca^2+^ Exit from Endoplasmic Reticulum and Mitochondrial Membrane Potential in Cultured Astrocytes

**DOI:** 10.1371/journal.pone.0126314

**Published:** 2015-05-08

**Authors:** Pavel Montes de Oca Balderas, Penélope Aguilera

**Affiliations:** 1 Unidad de Neurobiología Dinámica, Departamento de Neuroquímica, Instituto Nacional de Neurología y Neurocirugía, México City, México; 2 Laboratorio de Patología Vascular Cerebral, Instituto Nacional de Neurología y Neurocirugía, México City, México; University of São Paulo, BRAZIL

## Abstract

Astrocytes were long thought to be only structural cells in the CNS; however, their functional properties support their role in information processing and cognition. The ionotropic glutamate N-methyl D-aspartate (NMDA) receptor (NMDAR) is critical for CNS functions, but its expression and function in astrocytes is still a matter of research and debate. Here, we report immunofluorescence (IF) labeling in rat cultured cortical astrocytes (rCCA) of all NMDAR subunits, with phenotypes suggesting their intracellular transport, and their mRNA were detected by qRT-PCR. IF and Western Blot revealed GluN1 full-length synthesis, subunit critical for NMDAR assembly and transport, and its plasma membrane localization. Functionally, we found an *i*Ca^2+^ rise after NMDA treatment in Fluo-4-AM labeled rCCA, an effect blocked by the NMDAR competitive inhibitors D(-)-2-amino-5-phosphonopentanoic acid (APV) and Kynurenic acid (KYNA) and dependent upon GluN1 expression as evidenced by siRNA knock down. Surprisingly, the *i*Ca^2+^ rise was not blocked by MK-801, an NMDAR channel blocker, or by extracellular Ca^2+^ depletion, indicating flux-independent NMDAR function. In contrast, the IP_3_ receptor (IP_3_R) inhibitor XestosponginC did block this response, whereas a Ryanodine Receptor inhibitor did so only partially. Furthermore, tyrosine kinase inhibition with genistein enhanced the NMDA elicited *i*Ca^2+^ rise to levels comparable to those reached by the gliotransmitter ATP, but with different population dynamics. Finally, NMDA depleted the rCCA mitochondrial membrane potential (mΔψ) measured with JC-1. Our results demonstrate that rCCA express NMDAR subunits which assemble into functional receptors that mediate a metabotropic-like, non-canonical, flux-independent *i*Ca^2+^ increase.

## Introduction

Traditionally, the role of astrocytes in the CNS had been circumscribed to structural cells supporting neuronal functions, the cells in charge of carrying out the integration, processing and storage of information. However, research in the last decades has challenged this neurocentric view. Astrocytes are now known to secrete gliotransmitters, to express neurotransmitter receptors and more importantly, to form functional networks in which intracellular Ca^2+^ (*i*Ca^2+^) is critical for cell communication [1–8]. These properties support the tripartite synapse hypothesis [9]. Despite the fact that specific astrocyte functions were demonstrated in cognitive processing [10], other groups have neglected the role of astrocytes in synaptic activity, and in long- and short-term plasticity [11–13]. Thus, astrocytes’ role within the CNS is still a matter of debate [14].

The N-methyl-D-aspartate receptor (NMDAR) is a ionotropic glutamate (Glu) receptor with a critical role in the CNS. It is a hetero-tetramer of homo-dimers or a hetero–trimer formed by one or two GluN1 subunits associated with GluN2 (GluN2A-D) and/or GluN3 (GluN3A-B) subunits [15]. The GluN1 subunit is essential for NMDAR assembly, intracellular traffic and function because neither GluN2 or GluN3 subunits can exit the Endoplasmic Reticulum (ER) without associating with GluN1, which is required to mask their ER retention signals [16–21]. Thus, plasma membrane NMDAR’s can alternatively be constituted by GluN2 and/or GluN3 subunits but GluN1 subunits are obligatory.

NMDAR expression and canonical function in astrocytes has been demonstrated using acute isolated astrocytes or brain slices [5, 7, 22–25]; however, NMDAR function in cultured astrocytes is a matter of debate due to contradictory findings. Some authors have claimed unanimous agreement that cultured astrocytes are devoid of functional NMDAR, whereas other groups have found functional NMDAR in these cells [4, 24, 26–32].

The mitochondrial membrane potential (m0Δψ) is the voltage gradient across the inner mitochondrial membrane that results from net H^+^ outflow driven by the mitochondrial electron transport chain. mΔψ is crucial since several functions, including Ca^2+^ uptake, depend upon this voltage gradient [33, 34]. Mitochondria are known to act as an efficient Ca^2+^ buffer, and Ca^2+^ increase leads to the allosteric activation of some enzymes of the tricarboxylic acid cycle, boosting oxidative phosphorylation [33, 34].

In this work we demonstrate NMDAR expression and function in rCCA. Surprisingly, we found non-canonical flux-independent metabotropic-like NMDAR function that depletes the mΔψ.

## Materials and Methods

### Cortical astrocyte cultures

rCCA were prepared as described before [35] with modifications. Postnatal Wistar rats (*Rattus norvegicus*) were decapitated on day 0 accordingly to guidelines established by the Institutional Committee for Laboratory Animal Care and Use from the National Institute for Neurology and Neurosurgery that approved this study. Cortices (2–8) were dissected in cold Hank’s Balanced Salt Solution (HBSS) and dissociated. The cells were recovered, washed and seeded (2.5X10^4^ cells/cm^2^) in culture medium (DMEM with 10% FBS, 100 U/ml penicillin and 100 ng/ml streptomycin) into poly-D-lysine (PDL) tissue culture flasks. The medium was replaced after 24 h and every four days thereafter. At confluence (12–14 days), flasks were shaken to detach microglia and astrocytes were harvested with trypsin and seeded at 8X10^3^ cells/cm^2^. The cells were used for the indicated experiments 3–8 days later.

### Reagents and antibodies

Salts, reagents, NMDA, adenosine tri-phosphate (ATP), D(-)-2-amino-5-phosphonopentanoic acid (APV), Kynurenic acid (KYNA), Dizocilpine (MK-801), XestosponginC (XesC), Ryanodine (Ry), Genistein (GX) and carbonyl cyanide 3-chlorophenylhydrazone (CCCP) were from Sigma Chemical Co. (St. Louis, MO, USA). Cell culture media, supplements, master mix, Hoechst, 5,5’,6,6’-tetrachloro-1,1’,3,3’-tetraethylbenzim- idazolylcarbocyanine iodide (JC-1), Fluo-4 acetomethylester (Fluo-4-AM) and Lipofectamine 2000 were from Life Technologies (Carlsbad, CA, USA). Culture plates were from Costar (Tewksbury, MA, USA). Antibodies (Abs) against NMDAR subunits were purchased from Santa Cruz Biotechnology (Santa Cruz, CA, USA). Secondary Abs were from Jackson Immunochemicals (West Grove, PA, USA). Western Blot (WB) reagents were from Bio-Rad (Hercules, CA, USA), Enhanced Chemiluminiscent (ECL) reagents were from Thermo Scientific (Rockford, IL, USA), and Hybond and Hyperfilm were from GE Healthcare Amersham (Piscataway, NJ, USA). Tripure reagent and Protease Inhibitory cocktail were from Roche (Basel, CH). M-MLV reverse transcriptase was from Promega (Madison, WI, USA). Labeled Taq-Man probes for NMDAR subunits or 18S rRNA and siRNA were from Applied Biosystems (Foster City, CA, USA).

### Immunofluorescence (IF)

Cells seeded onto coverslips were washed with PBS, fixed with PBS-4% paraformaldehyde-4% sucrose on ice and then permeabilized with PBS-0.5% Tween-20. After washing, the cells were incubated with primary Ab (1 μg/ml) one hour in M1 buffer (140 mM NaCl, 20 mM HEPES, 1 mM Cl_2_Ca, 1 mM Cl_2_Mg, 5 mM KCl), washed and then with secondary Ab (46.6 ng/ml) one hour. Nuclei were labeled with Hoechst 33342 (10 μM), washed and mounted with homemade Mowiol-DABCO. *z*-stacks were acquired (60X N.A. 1.35 objective) in an Olympus IX-81 (Olympus Corporation) microscope with a Hamamatsu Orca Flash 2.8, Cell Sens Dimension software (Olympus Corporation) and mercury lamp illumination. Stacks were processed with software deconvolution algorithm.

### Quantitative RT-PCR (qRT-PCR)

Total RNA was extracted from rCCA using Tripure reagent according to manufacturer’s instructions. First strand cDNA was synthesized using 5 μg of total RNA, 50 ng degenerate primers and 200 U M-MLV reverse transcriptase. One μL of cDNA (dilution 1:10) was used as template for characterization of target genes. PCR reactions were performed at 95°C for 10 min and subjected to 40 cycles (92°C for 15 s and 60°C for 1 min) on a 7500 Real Time PCR System (Applied Biosystems, Foster City, CA, USA). Cycle threshold (Ct) values were determined by automated analysis using SDS Software 1.3.1. Data are averaged mRNA transcript relative levels normalized to 18S rRNA expression from triplicates and presented as 2^-ΔΔCT^ in accord with previous reports using normalized brain expression levels [29].

### Western Blot

Cells were washed with cold PBS, lysed with reducing sample buffer containing protease inhibitors and boiled 5 min. Lysates were resolved on SDS-polyacrylamide gels, and proteins were transferred to a nitrocellulose membrane. Protein detection was conducted by incubating with primary Ab (66 ng/ml) one hour, then with the appropriate secondary Ab (26 ng/ml) one hour and then revealed by ECL. The blot was stripped and re-probed after detection controls were performed.

### Intracellular calcium (*i*Ca^2+^) measurements

*i*Ca^2+^ determinations were performed as described previously [36] with modifications. rCCA were loaded for 45 min with 1 μM Fluo-4-AM, 0.02% pluronic acid in HBSS with 1.2 mM CaCl_2_, 400 μM MgSO_4_ and 440 μM MgCl_2_ (referred to as vehicle). Coverslips were transferred to a stage chamber and fluorescence emission was recorded with a 10X 0.4 N.A. objective with the microscope set up described above. Fluorescence was recorded with a 485/30 bandpass excitation filter, a 505 longpass dichroic mirror and a 510/50 bandpass emission filter. Time-lapse recordings were acquired at 20 frames per min (f/m). After 120 sec recording basal fluorescence with vehicle perfusion at 400 μl/min with or without inhibitors, NMDA, ATP or vehicle was perfused and recording continued for another 230 sec. Before the experiment, cells were treated 15–45 min with inhibitors that were constantly perfused during the recording. Time–lapses were analyzed with Cell Sens Dimension software. Background noise was subtracted and oval regions of interest (ROI) were drawn around each cell soma. Labeling phenotypes showing bright cytoplasmic puncta were discarded. The mean fluorescence intensity (F) was obtained for each ROI’s and *i*Ca^2+^ responses were evaluated by calculating the relative fluorescence change (ΔF/F_0_) with the formula ΔF/F_0_ = (F*t*_*x*_-F_0_)/F_0_, where F*t*_*x*_ is F at time *x* and F_0_ is the average fluorescence during the first 50 sec. Data are presented as average response traces with cells obtained from at least three different cultures. The integrated relative fluorescence change ∫(ΔF/F_0_) for each cell was calculated as the sum of normalized ΔF/F_0_ values between 0 and 350 sec. ∫(ΔF/F_0_) values for the cell populations tested are presented as distribution histograms for each experiment. The number of cells (*n*), experiments and numerical descriptors for averaged responses and distribution histograms are found in Table 1. For experiments in Ca^2+^-free conditions cells were pre-incubated for at least 15 min and up to 45 min before recording.

**Table 1:** Data summaries from averaged responses and distribution histograms in Figs 4–7.

	vehicle	NMDA	APV- NMDA	KYNA- NMDA	siC-NMDA	siGrin-NMDA	MK-801-NMDA	Ca^++^- free HBSS	XesC-NMDA	Ry-NMDA	GX-NMDA	ATP
***# of cells(n)/# of experiments***	520/8	374/8	176/4	123/4	328/9	291/10	366/10	464/10	435/10	84/4	368/7	518/7
**Averaged responses peak height±S.D.**	n.a.	0.42±0.45	0.008±0.05	0.06±0.22	0.34±0.64	0.07±0.36	0.31±0.57	0.67±0.875	0.19±0.5	0.22±0.32	0.83±0.6	1.09±0.93
**Average ∫(ΔF/F**_**0**_ **)±SEM**	**6.2±0.2**	**20.5±0.9**	**10.7±0.4**	**9.2±0.4**	**14±0.8**	**8±0.5**	**21.9±1**	**24.6±0.9**	**8.8±0.4**	**12.1±1.4**	**24.8±0.8**	**26.8±0.7**
**% above RTV (responsive cells)**	**4**	**53.5**	**19.3**	**8.9**	**35.4**	**12.7**	**43.7**	**56**	**14.3**	**27.4**	**73.9**	**76.5**
**% below RTV (non-responsive cells)**	**96**	**46.5**	**80.7**	**91.1**	**64.6**	**87.3**	**56.3**	**44**	**85.7**	**72.6**	**26.1**	**23.5**
**% response normalized to NMDA response**	***0*.*0***	***100*.*0***	***31*.*1***	***20*.*9***	***54*.*2***	***12*.*3***	***109*.*6***	***128*.*3***	***17*.*9***	***41*.*1***	***129*.*9***	***143*.*5***
**Kruskall-Wallis test vs. NMDA treated cells**	*p≤0*.*01*	*n*.*a*.^*1*^	*p≤0*.*01*	*p≤0*.*01*	*p≤0*.*01*	*p≤0*.*01* ^***3***^	*n*.*s*.^*2*^	*n*.*s*.^*2*^	*p≤0*.*01*	*p≤0*.*01*	*p≤0*.*01*	*p≤0*.*01* ^*4*^

^*1*^ *not-applicable*

^*2*^ *non significant*

^*3*^ *vs*. *siC transfected cells*

^*4*^ *vs*. *GX-NMDA treated cells*.

### Cell transfection

Astrocytes were transfected with Lipofectamine 2000 according to the manufacturer’s conditions. Briefly, Lipofectamine 2000 and siRNA were mixed and incubated in Opti-MEM for the suggested times, this mix was added to cells in fresh antibiotic-free medium. Transfected cells were used for *i*Ca^2+^ measurements 24 h after transfection.

### Mitochondrial Membrane Potential (mΔψ)

rCCA were labeled with 600 ng/ml JC-1 for 15 min at 37°C. The medium was replaced and time-lapse recordings were made with a 10X 0.4 N.A. objective and the same microscope set up described above with a Hammamatsu Orca R2 CCD and homemade white LED illumination. Time-lapse recordings were acquired at 1 f/min. JC-1 orange-red fluorescence was recorded with a 562/35 bandpass excitation filter, a 600 longpass dichroic mirror and a 610 longpass emission filter. JC-1 green fluorescence was recorded with a 485/30 bandpass excitation filter, a 505 longpass dichroic mirror and a 510/50 bandpass emission filter. After obtaining basal readings in 3 frames (*t0*-*t2*), cells were treated with 1 mM NMDA or vehicle and recorded for 9 min; then, rapid mitochondrial depolarization was induced with 10 μM CCCP. Time–lapse recordings were analyzed with Cell Sens Dimension software. Background noise was subtracted and ROIs were drawn around each cell soma. The average fluorescence intensity for green (*x*F_*i*_g) and orange-red (*x*F_*i*_or) channels were obtained for each ROI and mΔψ was calculated as ratios [mΔψ = (*x*F_*i*_or)/(*x*F_*i*_g)] (from now on referred to as mΔψ). Data are presented as average mΔψ±s.e.m. Cell-by-cell mΔψ change rates for some time points were evaluated and are presented as distribution histograms.

### Statistical analysis

Statistical differences in *i*Ca^2+^ experiments were analyzed by the Kruskal-Wallis test of ∫(ΔF/F_0_) distribution using Graphpad Prism 5.01 (Graphpad Software Inc.). Significance is claimed with p≤0.01. For mΔψ experiments, statistical differences at each time point of the time-lapse were determined by the student’s t-test with Excel (Microsoft). Significance is claimed with p≤0.05 or p≤0.01. Cell-by-cell change rates for mΔψ are presented as average ± s.e.m.

## Results

### NMDAR subunits expression

**Fig 1:**
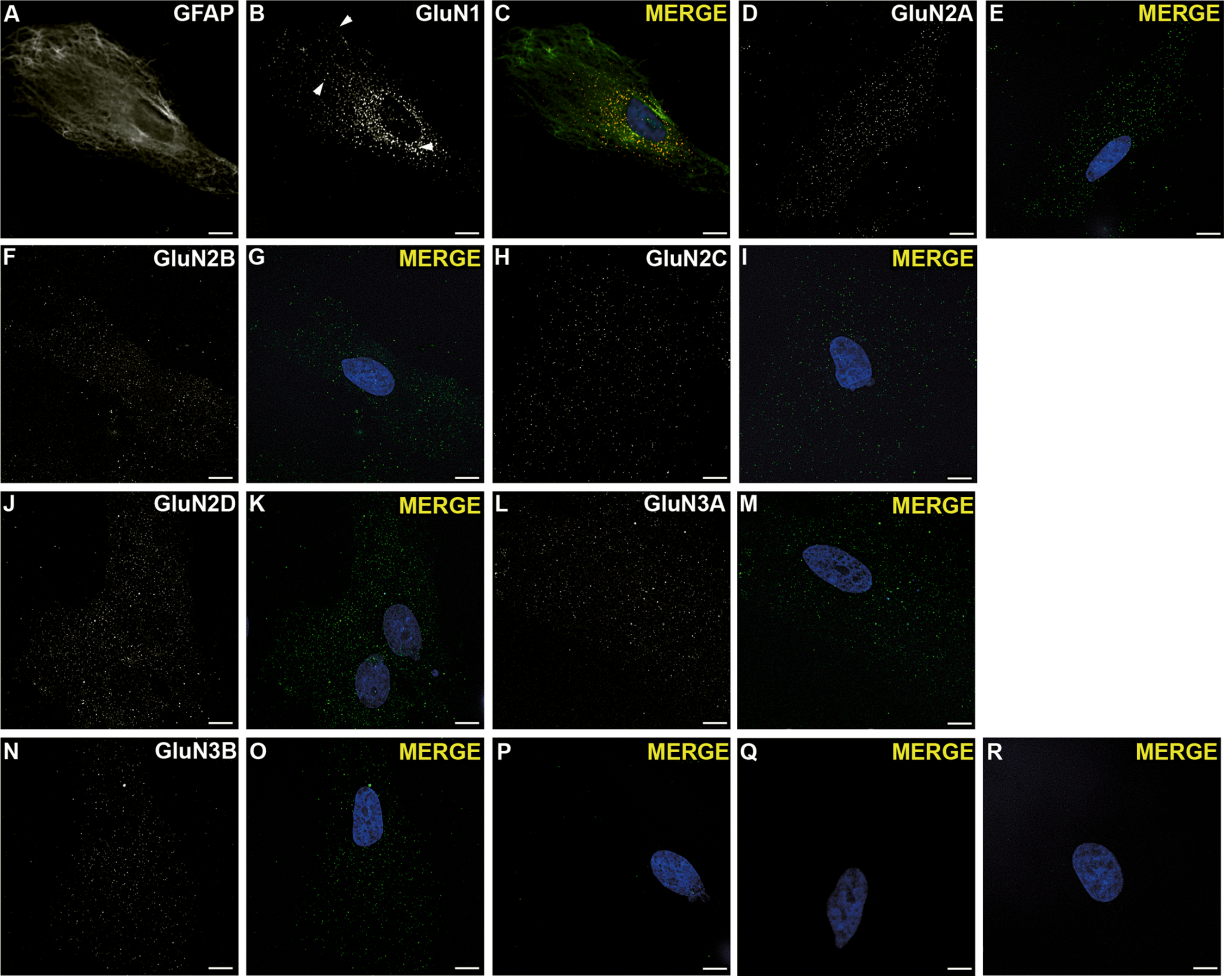
NMDAR subunits in permeabilized rCCA. (**A**) GFAP positive cells were double labeled with a goat polyclonal Ab against the GluN1 IC domain (**B**) that showed near plasma membrane (top arrow), intracellular (middle arrow) and perinuclear puncta (bottom arrow). (**C)** Merged image with stained nucleus. (**D**) GluN2A IF with a goat polyclonal Ab. (**E**) Merged image with stained nucleus. (**F**) GluN2B IF with a mouse monoclonal Ab. (**G**) Merged image with stained nucleus. (**H**) GluN2C IF with a polyclonal rabbit Ab. (**I**) Merged image with stained nucleus. (**J**) GluN2D IF with a monoclonal mouse Ab. (**K**) Merged image with stained nucleus. (**L**) GluN3A IF with a goat monoclonal Ab. (**M**) Merged image with stained nucleus. (**N**) GluN3B IF with a polyclonal rabbit Ab. (**O**) Merged image with stained nucleus. These phenotypes were not observed in cells without primary Ab but with identical secondary Ab concentrations against goat IgG (**P**); rabbit IgG (**Q**); and mouse IgG (**R**). In all images the bottom slice from a *z* stack after blind deconvolution processing from a representative cell from 3 independent experiments is shown. Reference bar = 10 μm.

We initially characterized the purity of our rCCA by GFAP staining and found that >95% of cells were positive (S1 Fig). Then we tested whether these cells expressed GluN1 through double IF. As shown (Fig 1A–1C), cells presented labeling with a polyclonal Ab against the GluN1 IC domain, with puncta in the cytoplasmic and perinuclear areas and near plasma membrane (arrows, Fig 1B). This phenotype was not observed in control experiments with identical secondary Ab concentration but without primary Abs (Fig 1P).

In addition, rCCA were labeled by Abs against all NMDAR subunits: a polyclonal Ab against GluN2A EC domain; a monoclonal Ab against the GluN2B EC domain; a polyclonal Ab against the GluN2C EC domain; a monoclonal Ab against the GluN2D EC domain; a polyclonal Ab against the GluN3A EC domain; and a polyclonal Ab against the GluN3B EC domain (Fig 1D–1O). Phenotypes labeled with these Abs showed puncta throughout cell soma that were not observed in control experiments (Fig 1P–1R). These observations strongly suggested that NMDAR subunits are synthesized in rCCA, and more importantly, that the NMDAR subunits are transported intracellularly.

**Fig 2:**
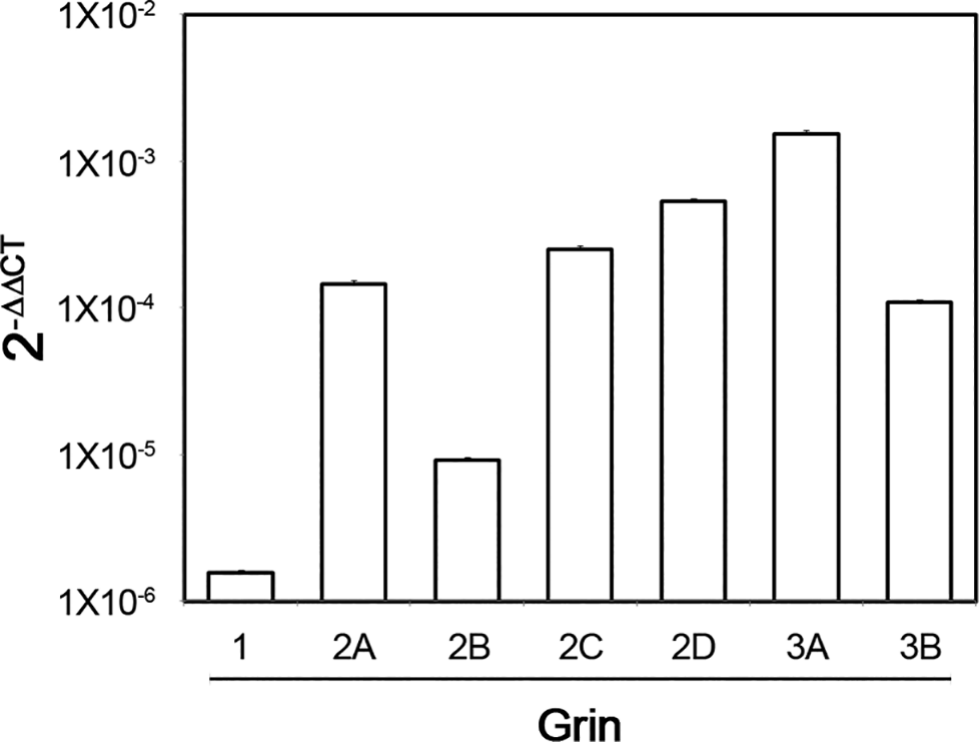
NMDAR subunit mRNA expression. The bars represent 2^-ΔΔ Ct^ averages ± s.d. of triplicates from one representative experiment of three independent experiments with 18S rRNA as reference gene.

We next investigated NMDAR subunit mRNA expression by qRT-PCR and found mRNA expression of all NMDAR subunits (Fig 2). These data confirmed that the genes for all NMDAR subunits are expressed in rCCA, as hinted at by the IF experiments. Furthermore, these results suggested differences in the transcription levels of the genes for these subunits, as follows: Grin3A>Grin2A≈Grin2C≈Grin2D≈Grin3B>Grin2B≈Grin1. Negative (without DNA) and positive (DNA from rat brain) controls for RT-PCR reactions were performed in parallel with these experiments (S2 Fig).

### Full length GluN1 expression and cell membrane localization

**Fig 3:**
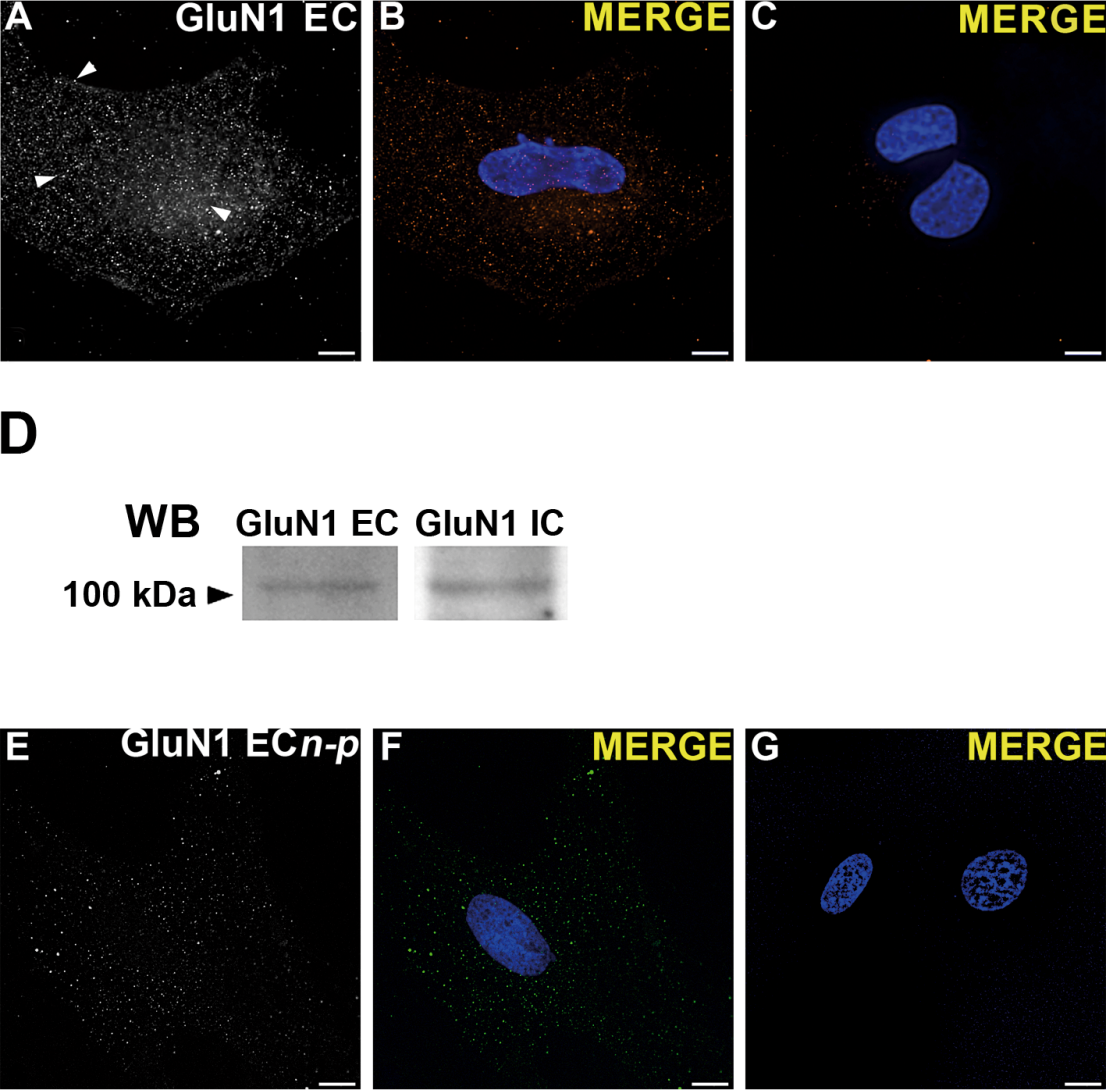
Full-length GluN1 expression and cell membrane localization. (**A**) GluN1 IF in permeabilized rCCA with a polyclonal Ab against its EC domain showing puncta near the plasma membrane (top arrow), intracellular (middle arrow) and perinuclear (bottom arrow). (**B**) Merged image with stained nucleus. This phenotype was not observed in cells without primary Ab but with identical secondary Ab concentrations (**C**). (**D**) WB of whole cell lysates with Abs against GluN1 EC (left panel) and IC (right panel) domains in the same blot after stripping. A band of ≈115 kDa corresponding to full-length GluN1 was detected with both Abs. One representative experiment is shown from at least three performed independently. (**E**) GluN1 IF in non-permeabilized rCCA with a polyclonal Ab against its EC domain. (**F**) Merged image with stained nucleus. This phenotype was not observed in non-permeabilized cells without primary Ab but with identical secondary Ab concentrations (**C**). In all images the bottom slice from a z-stack after blind deconvolution processing from a cell representative of 3 independent experiments is shown. Reference bar = 10 μm.

We next tested GluN1 full-length expression since, considering its relevance for NMDAR assembly, transport and function, its truncated expression could result in a non-functional NMDAR. In our IF experiments with a polyclonal Ab against the GluN1 EC N-terminal domain, we observed a phenotype characteristic of transmembrane molecules, with puncta distributed throughout the cytoplasm, perinuclearly and near the plasma membrane (arrows, Fig 3A and 3B). No labeling was observed without primary Ab (Fig 3C). This result, together with the GluN1 IC domain labeling described above suggested its full-length expression. We further assessed this possibility by WB. Our results showed that a ≈115 kDa band, corresponding to full-length GluN1 molecular mass (M_r_), was recognized by Abs against both the C- and N- terminal domains (Fig 3D). Importantly, both Abs detected this band in the same blot after stripping and performing detection controls, thus identifying GluN1 by its M_r_.

These results demonstrated that rCCA express full-length GluN1, making feasible NMDAR assembly in the ER and its transport to other cellular compartments. Therefore, we assayed for GluN1 localization at the plasma membrane in non-permeabilized rCCA. As observed in Fig 3E and 3F, the Ab against its EC N-terminal domain showed extracellular membrane puncta that were not observed in control experiments without primary Ab (Fig 3G), confirming plasma membrane localization of GluN1. Together, these findings make it conceivable that NMDAR assemblies exit the ER and reach the plasma membrane where they may be functional.

### NMDA elicits an *i*Ca^2+^ increase that is blocked by NMDAR competitive inhibitors

**Fig 4:**
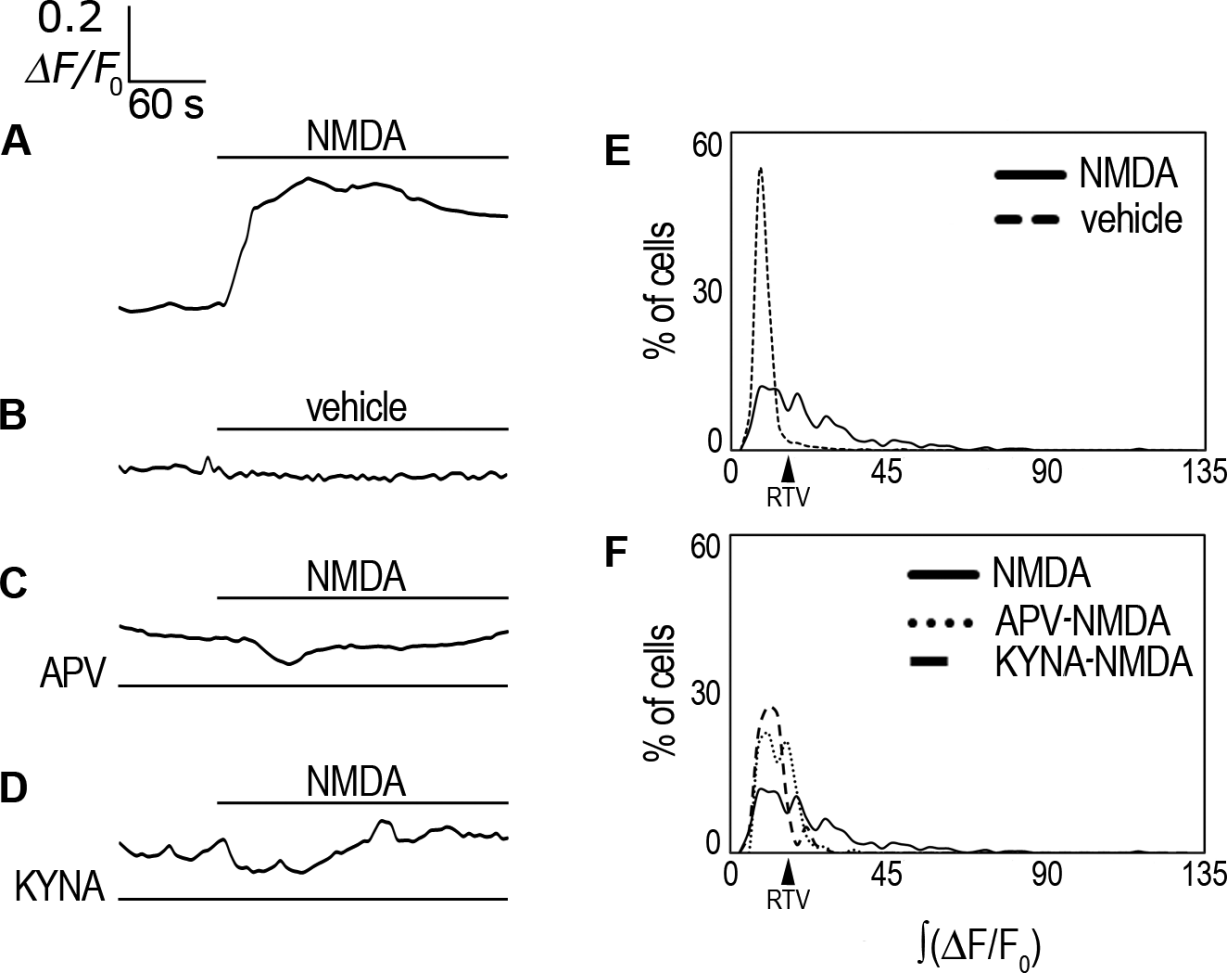
NMDA effect on *i*Ca^2+^. (**A**) ΔF/F_0_ averaged response in Fluo-4-AM-labeled rCCA perfused with 1 mM NMDA. (**B**) ΔF/F_0_ averaged response in Fluo-4-AM-labeled rCCA perfused with vehicle alone. (**C**) ΔF/F_0_ averaged response in Fluo-4-AM-labeled rCCA perfused with NMDA in the presence of APV or (**D**) KYNA. Line above traces indicates perfusion with 1 mM NMDA or vehicle after 120 sec basal recording. Line below (if applicable) indicates the inhibitor used throughout the recording time. (**E**) ∫(ΔF/F_0_) distribution histogram for cell population responses in NMDA and vehicle conditions. (**F**) ∫(ΔF/F_0_) distribution histogram for cell population responses in APV and KYNA conditions; the NMDA treated population distribution is included for comparison. The RTV value is indicated by the black arrowhead (see text). Statistical analyses for these distributions with the number of cells and the number of experiments are shown in Table 1. Representative images of *i*Ca^2+^ responses are shown in S8 Fig.

We then tested for NMDAR function by measuring *i*Ca^2+^ after NMDA treatment using the Ca^2+^ sensor Fluo-4-AM. We found that 1 mM NMDA induced a ≈0.4 0ΔF/F_0_ maximal increase of averaged response (Table 1; Fig 4A), whereas 100 μM NMDA had no effect (S3 Fig). In contrast, vehicle alone did not exhibit this effect (Table 1; Fig 4B). The rCCA response to NMDA was given by an increase of the average ∫(ΔF/F_0_) (Table 1). In addition, we determined a reference threshold value (RTV) to examine population dynamics, considering the vehicle-treated population average ∫(ΔF/F_0_) plus twice its s.d. (RTV = 14.8; Table 1). Cells below this value were considered non-responsive. We found that over tenfold more cells presented a ∫(ΔF/F_0_) value above the RTV after perfusion with NMDA than with vehicle alone (Table 1). This is observed in the distribution histograms describing the population dynamics (Fig 4E). These histograms also revealed a diversity of *i*Ca^2+^ cell responses after NMDA treatment, with a main peak of cells (≈35%) that displayed a modest *i*Ca^2+^ increase just above the RTV, and another ≈18% of cells distributed in additional peaks, indicating heterogeneous, larger responses. These experiments strongly suggested that rCCA possess functional NMDARs.

Next, we investigated the response specificity using two competitive inhibitors of the NMDAR: APV, a Glu site inhibitor of GluN2 subunits, and KYNA, a competitive inhibitor of the glycine (Gly) site in GluN1 subunit. Our experiments showed that APV (100 μM) blocked the averaged *i*Ca^2+^ response of rCCA to NMDA (Table 1; Fig 4C). ∫(ΔF/F_0_) analysis showed that the APV blockade was significant and reached almost 70% with only 19.3% of cells above the RTV (Table 1; Fig 4F). On the other hand, KYNA (20 μM) also inhibited the averaged *i*Ca^2+^ response of rCCA to NMDA (Table 1; Fig 4D). ∫(ΔF/F_0_) examination showed that KYNA significantly reduced by 80% the rCCA response to NMDA with only 8.9% of cells above the RTV (Table 1; Fig 4F).

Taken together these results demonstrated that rCCA express functional NMDARs that, after ligand binding, mediate *i*Ca^2+^ increase, and they rule out unspecific effects associated with the NMDA concentration employed.

### Grin1 knock down inhibits rCCA response to NMDA

**Fig 5:**
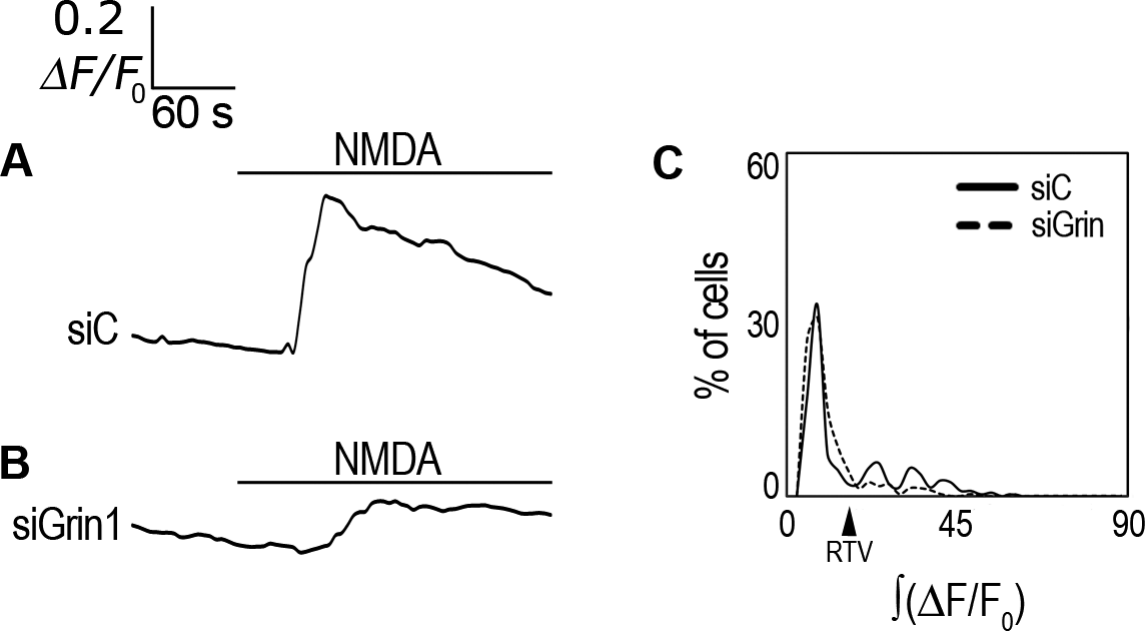
Grin1 knock down effect on *i*Ca^2+^ response to NMDA. (**A**) F/F_0_ averaged response in Fluo-4-AM-labeled rCCA transfected with a control siRNA (siC) and perfused with 1 mM NMDA. (**B**) ΔF/F_0_ averaged response in Fluo-4-AM-labeled rCCA transfected with a Grin1 siRNA (siGrin1) and perfused with 1 mM NMDA. Line above traces indicates perfusion with 1 mM NMDA after 120 sec basal recording. (**C**) ∫(ΔF/F_0_) distribution histograms for cell population responses after siRNA transfection. The RTV value is indicated by the black arrowhead (see text). Statistical analyses for these distributions with the number of cells and the number of experiments are shown in Table 1. Representative images from *i*Ca^2+^ responses are shown in S8 Fig.

To further test NMDA specificity we analyzed the response of rCCA to NMDA after GluN1 gene (Grin1) knock down. Initially, we determined that the highest knock down efficiency (>55%) and cell viability were obtained with 2 μl/ml Lipofectamine and 166 nM siRNA (S4 Fig) and the used these conditions for further experiments. When a control siRNA (siC) was transfected, rCCA displayed an averaged response to NMDA similar to that of non-transfected cells, i.e., with a maximal increase in ΔF/F_0_ = 0.34, but with a larger decrease with time (Table 1; Fig 5A). In contrast, GluN1 knock down reduced the averaged response of rCCA to NMDA (Table 1; Fig 5B). ∫(ΔF/F_0_) analysis showed that this reduction was statistically significant and reached 76% when compared with siC transfected cells and only 12.7% of cells had a response above the RTV (Table 1). These experiments confirmed the specificity of the NMDA effect, but also showed that GluN1 expression is critical to acquire a functional NMDAR in these cells. Notably, ∫(ΔF/F_0_) analysis showed that, although the maximal averaged response of siC-transfected cells was similar to that of untransfected cells, siC transfection significantly decreased ∫(ΔF/F_0_) by 46% when compared with untransfected cells (Table 1). This was given by the decrease of *i*Ca^2+^ by the end of the recording time and 35.4% of cells above the RTV (Table 1, Fig 5C). Unexpectedly, we also found that the transfection protocol itself resulted in a large population of non-responsive cells with both siRNAs (Fig 5C; see discussion).

### NMDA effect on *i*Ca^2+^ is NMDAR flux-independent but IP_3_R dependent

**Fig 6:**
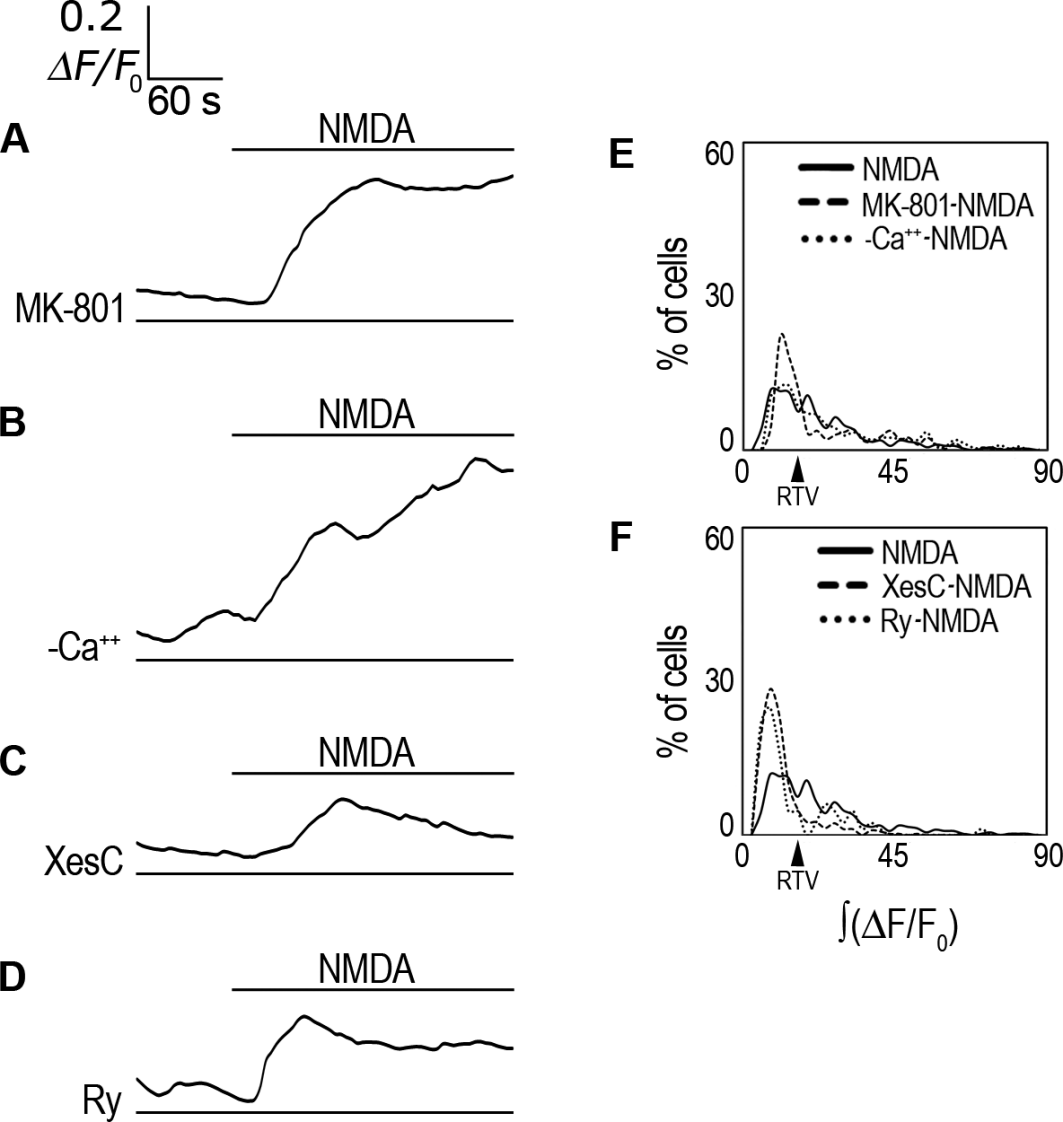
Analysis of *i*Ca^2+^ source. (**A**) ΔF/F_0_ averaged response in Fluo-4-AM-labeled rCCA perfused with 1 mM NMDA in the presence of MK-801. (**B**) ΔF/F_0_ averaged response in Fluo-4-AM-labeled rCCA perfused with 1 mM NMDA under extracellular Ca^2+^-free conditions. (**C**) ΔF/F_0_ averaged response in Fluo-4-AM-labeled rCCA perfused with 1 mM NMDA in the presence of XestosponginC. (**D**) ΔF/F_0_ averaged response in Fluo-4-AM-labeled rCCA perfused with 1 mM NMDA in the presence of Ryanodine. Line above traces indicates perfusion with 1 mM NMDA after 120 sec basal recording. Line below (if applicable) indicates the inhibitor used throughout the recording time. (**E**) ∫(ΔF/F_0_) distribution histogram for cell population responses in MK-801 and extracellular Ca^2+^-free conditions, NMDA distribution is included for comparative purposes. (**F**) ∫(ΔF/F_0_) distribution histogram for cell population responses in XestosponginC and Ryanodine conditions; the NMDA treated population distribution is included for comparison. The RTV value is indicated by the black arrowhead (see text). Statistical analysis for these distributions with the number of cells and the number of experiments are shown in Table 1. Representative images from *i*Ca^2+^ responses are shown in S8 Fig.

To further characterize the NMDA effect, we used MK-801, an irreversible NMDAR channel blocker. To our surprise, MK-801 (10 μM) did not block the averaged response to NMDA (Table 1; Fig 6A), and ∫(ΔF/F_0_) examination showed no significant difference with NMDA perfused cells (Table 1). MK-801 only slightly increased the number of non-responsive cells by 10% (Fig 6E; Table 1). This result suggested that the NMDAR in rCCA function in a non-canonical, Ca^2+^ flux-independent manner, as reported previously by some groups [37–43] (see discussion). We further tested this possibility by performing experiments in Ca^2+^-free conditions. Consistent with this interpretation, extracellular Ca^2+^ depletion did not block the NMDA effect (Table 1; Fig 6B), and ∫(ΔF/F_0_) analysis showed no difference with cells treated with NMDA in the presence of extracellular Ca^2+^ (Table 1, Fig 6E). These results strongly suggested that the NMDARs function in a flux-independent manner; otherwise *i*Ca^2+^ response with MK-801 or without extracellular Ca^2+^ should be blocked, as expected for the canonical NMDAR function.

We then examined whether the *i*Ca^2+^ rise elicited by NMDA could due to Ca^2+^ exit from the ER. We used XestosponginC (XesC), an inhibitor of the Inositol trisphosphate (IP_3_) receptor (IP_3_R) in the ER that mediates Ca^2+^ exit. XesC (100 nM) blocked the averaged *i*Ca^2+^ response of rCCA to NMDA (Table 1; Fig 6C), and ∫(ΔF/F_0_) analysis showed that this inhibition was significant (Table 1). We also tested the effect of Ryanodine (Ry), an inhibitor of the Ryanodine receptor (RyR) that mediates Ca^2+^ exit from the ER after Ca^2+^ activation, a mechanism termed Ca^2+^-induced Ca^2+^ release (CICR). Ry (50 μM) reduced the averaged *i*Ca^2+^ response of rCCA to NMDA (Table 1; Fig 6D), and ∫(ΔF/F_0_) analysis showed that this reduction was significant although to a lower extent (Table 1, Fig 6F).

Taken together these results demonstrate that the rCCA response to NMDA is elicited by a metabotropic-like NMDAR flux-independent *i*Ca^2+^ rise that involves Ca^2+^ release from the ER mediated mainly by the IP_3_R but also by the RyR.

### Tyrosine kinase inhibition enhances *i*Ca^2+^ response to NMDA

**Fig 7:**
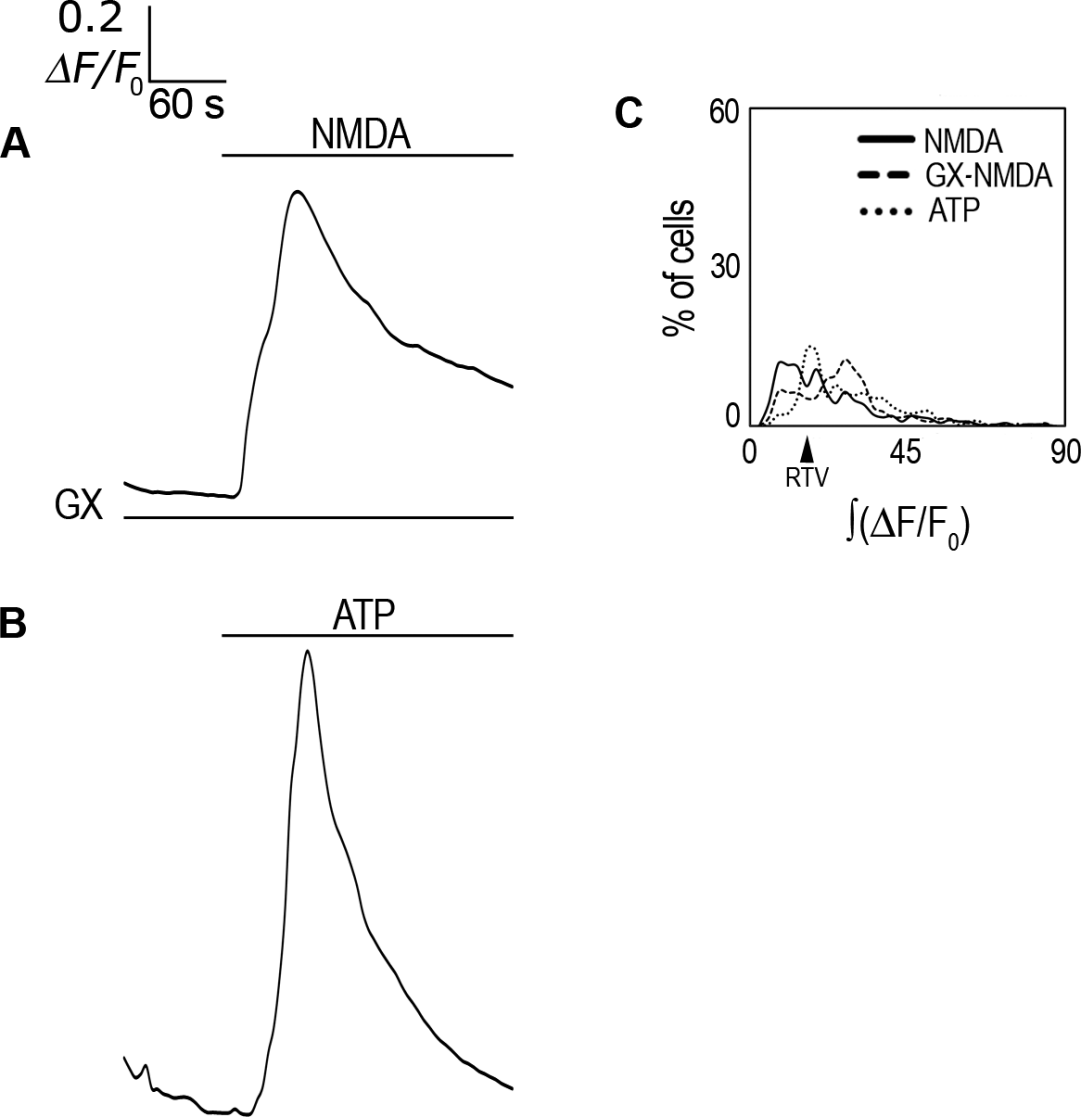
Genistein effect on *i*Ca^2+^ response to NMDA and ATP response. (**A**) ΔF/F_0_ averaged response in Fluo-4-AM-labeled rCCA perfused with 1 mM NMDA in the presence of genistein (GX). (**B**) ΔF/F_0_ averaged response in Fluo-4-AM-labeled rCCA perfused with ATP. Line above traces indicates perfusion with 1 mM NMDA after 120 sec basal recording. Line below (if applicable) indicates the inhibitor used throughout the recording time. (**C**) ∫(ΔF/F_0_) distribution histogram for cell population responses in genistein and ATP conditions; the NMDA treated population distribution is included for comparison. The RTV value is indicated by the black arrowhead (see text). Statistical analysis for these distributions with the number of cells and the number of experiments are shown in Table 1. Representative images from *i*Ca^2+^ responses are shown in S8 Fig.

Tyrosine kinases activity is known to regulate NMDAR function [15]; thus we investigated whether their inhibition could regulate the *i*Ca^2+^ response of rCCA to NMDA. Interestingly, Genistein (GX) (10 μM), a tyrosine kinase inhibitor, doubled the maximal averaged response to NMDA, eliciting a transient peak that reached ≈0.8 ΔF/F_0_ and did not returned to basal levels by the end of the recording (Table 1; Fig 7A). ∫(ΔF/F_0_) examination showed that this increase was significant (Table 1). Interestingly, cells above the RTV were distributed in a bimodal fashion, with a main peak containing ≈66% of cells and only 8% of cells with larger responses, contrasting with the heterogeneous responses evoked by NMDA alone (Fig 7C; Table 1; see discussion). These experiments indicated that NMDAR activity that elicits Ca^2+^ release from the ER is subject to tyrosine kinase down-regulation.

We then compared the *i*Ca^2+^ response to NMDA, with (Fig 7A) or without GX (Fig 4A), with the response to ATP, a well-known gliotransmitter that releases *i*Ca^2+^ from intracellular pools in cultured astrocytes [44, 45]. ATP (100 μM) showed a maximal averaged response that reached 1.1 ΔF/F_0_ (Table 1; Fig 7B), 2.6 times larger than the response to NMDA, and ≈30% larger than the response achieved with NMDA and GX together (GX-NMDA). ∫(ΔF/F_0_) analysis showed that the response to ATP was significantly different from that to NMDA alone (Table 1). In addition, the response to ATP was transient, returning to basal levels by the end of the recording time, kinetics that differed from the response to NMDA. The ATP and GX-NMDA responses also presented different kinetics because, as described above, the GX-NMDA response did not return to basal levels (Fig 7A). ∫(ΔF/F_0_) analysis showed that the ATP response was non-significantly different from the GX-NMDA response (Table 1). Nevertheless, their distribution histograms showed differences, because ATP left a smaller proportion of non-responsive cells than did GX-NMDA (Fig 7C; Table 1). Furthermore, GX-NMDA induced a larger *i*Ca^2+^ rise than ATP in a considerable proportion of cells, as evidenced by the shift in their main peaks (Fig 7C).

These comparisons demonstrated that NMDA and the well-known gliotransmitter ATP both evoke an *i*Ca^2+^ rise, but that these responses differ in their maximal response and duration. Moreover, these comparisons demonstrated that despite the similar (ΔF/F_0_) and ∫(ΔF/F_0_) values of the ATP and GX-NMDA responses, their population dynamics were different. Here, it is important to note that analysis of the ∫(ΔF/F_0_) distribution histograms from averaged responses that were not significantly different showed meaningful differences; thus these analyses may be a useful tool for examining population-based studies.

### mΔψ depletion by NMDA

**Fig 8:**
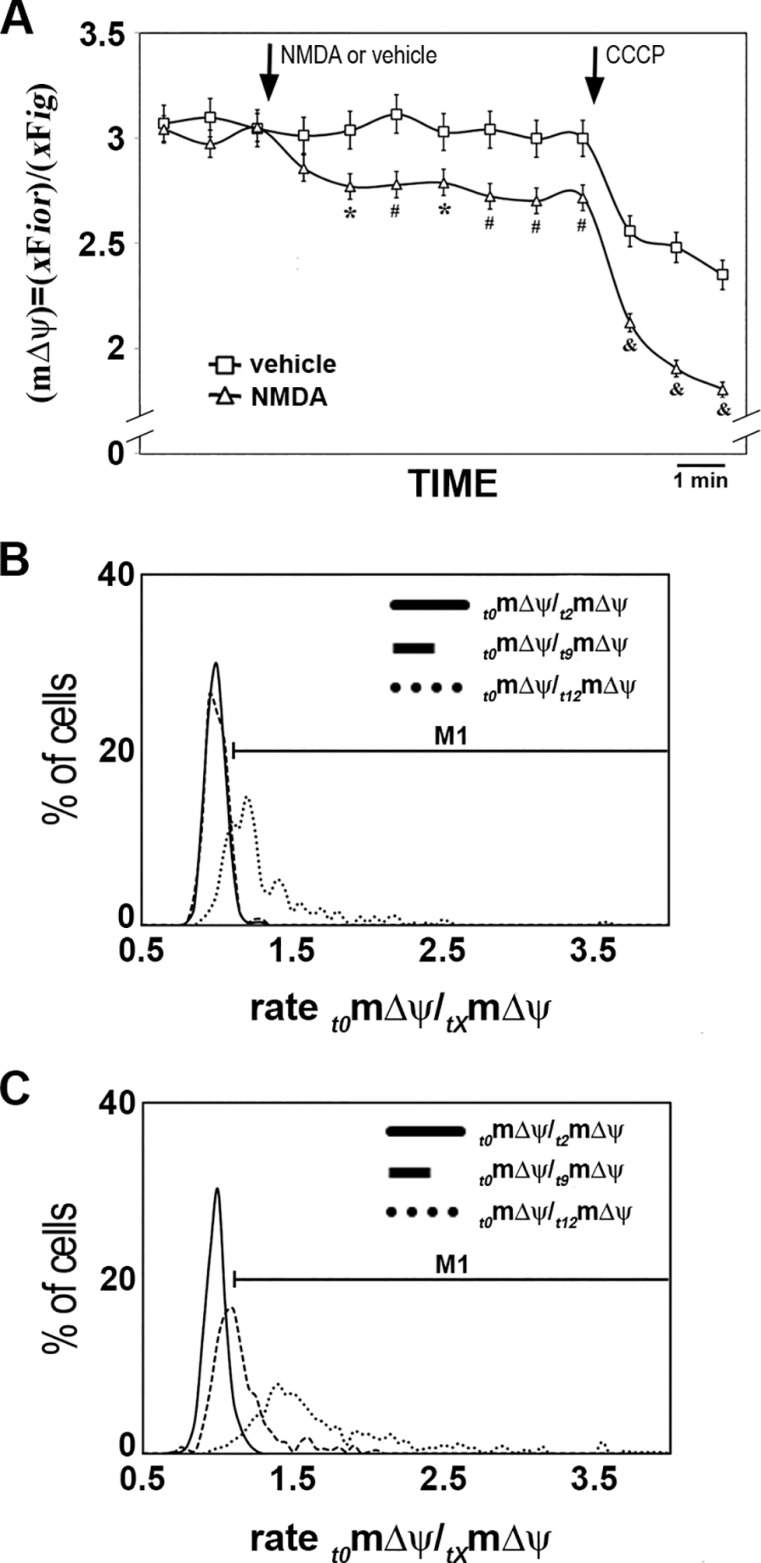
NMDA effect on mΔψ. (**A**) mΔψ time lapses of vehicle-(squares) and 1 mM NMDA-(triangles) treated rCCA labeled with JC-1. The three initial frames measured basal mΔψ. After basal recording, NMDA or vehicle was added as indicated by the arrow, and seven more frames were acquired. Finally, CCCP was added (arrow) as positive control for depolarization and three more frames were acquired. Distribution histograms for cell-by-cell change rate analysis for vehicle-(**B**) or NMDA-(**C**) treated rCCA at three different timepoints. Rates were calculated for each cell in vehicle and NMDA conditions between their initial (*t0*) mΔψ and three subsequent time points: at *t2* in basal conditions (_*t0*_mΔψ/_*t2*_mΔψ; line), at *t9* after NMDA treatment (_*t0*_mΔψ/_*t9*_mΔψ; dashed line), and at *t12* after CCCP treatment (_*t0*_mΔψ/_*t12*_mΔψ; dotted line). The M1 line above the histograms indicates the range used as reference to claim mΔψ depolarization. Data summaries for these histograms are presented in Table 2. (* = p≤0.05; # = p≤0.01; & = p≤0.001; student’s t-test).

We then investigated the NMDA effect on mΔψ, considering the role of mitochondria as *i*Ca^2+^ buffer. We found that NMDA treatment (1 mM) significantly reduced mΔψ (12 experiments; n = 570 cells from 3 cultures; student’s t-test; p≤0.05 or p≤0.01) in comparison with vehicle-treated cells (6 experiments; n = 305 cells from 3 cultures; Fig 8A), whereas 100 ΔM NMDA had no effect (S5 Fig). The maximal NMDA effect reached ≈25% of the maximal depolarization observed with CCCP. Notably, we observed that CCCP depleted mΔψ more dramatically after NMDA treatment than after vehicle treatment (see discussion).

**Table 2:** Data summaries from rate distribution histograms in Fig 8.

		average cell rate ±S.D.	% cells >S.D. (Depolarized^2^)	% cells <S.D.(Polarized)	% cells within S.D(Non-responsive)
_***t0***_ **m**Δψ**/**_***t2***_ **m**Δψ	VEHICLE^3^ **(basal)**	**1.019±0.069** ^**1**^	14.75	14.43	70.82
	EXPERIMENTAL^4^ **(basal)**	**1.012±0.075** ^**1**^	14.33	19.41	66.26
_***t0***_ **m**Δψ**/**_***t9***_ **m**Δψ	VEHICLE **(7 min basal)**	1.022±0.075	17.38	15.09	67.54
	EXPERIMENTAL **(7 min NMDA 1mM)**	1.169±0.199	64.34	4.72	30.94
_***t0***_ **m**Δψ**/**_***t12***_ **m**Δψ	VEHICLE **(3 min CCCP)**	1.342±0.325	87.87	0.66	11.48
	EXPERIMENTAL **(3 min CCCP)**	1.733±0.511	98.25	0.18	1.57

^1^these mean cell rate values ±S.D. were used as reference thresholds to determine mΔψ increase or decrease (see text for details).

^2^(M1 in Fig 8)

^3^6 experiments; n = 305 cells from three cultures

^4^12 experiments; n = 512 cells from three cultures

We next analyzed the NMDA effect on mΔψ at the individual cell level, because responses were heterogeneous, as reported previously [46, 47]. For this purpose, we calculated the mΔψ change rate for each cell, for initial mΔψ at *t0* (_t0_mΔψ) and three other time points: at *t2* (_t2_mΔψ) during basal conditions; at *t9* (_t9_mΔψ) after NMDA application; and at *t12* (_t12_mΔψ) after CCCP treatment. These change rates were plotted on distribution histograms for vehicle (Fig 8B) and NMDA-treated cells (Fig 8C), and data summaries are presented in Table 2. These rates allowed us to estimate the number of cells that underwent mΔψ depolarization (if its rate is larger than the population average at _t0_mΔψ/_t2_mΔψ+s.d; M1 in Fig 8B and 8C) or hyperpolarization (if its rate is smaller than the population average at _t0_mΔψ/_t2_mΔψ-s.d.), whereas cells within this range were considered non-responsive (Table 2). For the _t0_mΔψ/_t2_mΔψ rate (basal conditions), vehicle- and NMDA-treated cells showed similar distributions (Table 2; Fig 8B and 8C). In contrast, the average _t0_mΔψ/_t9_mΔψ rate (post NMDA) increased in NMDA-treated cells (Table 2; Fig 8B and 8C), but not in vehicle-treated cells that distributed similarly to basal conditions (Fig 8B and 8C; Table 2). Finally, after CCCP treatment, average _t0_mΔψ/_t12_mΔψ rates for NMDA- and vehicle-treated cells were larger than for cells in basal conditions (_t0_mΔψ/_t2_ mΔψ), and that of NMDA-treated cells was larger than that of vehicle-treated cells. These results confirmed functional NMDAR expression by rCCA and demonstrated that its activation regulates mitochondrial function.

## Discussion

Previous studies have reported functional, canonical ionotropic NMDARs in astrocytes using brain slices or acute tissue disaggregation [5, 7, 22–25], models that allow manipulations only shortly after cell or tissue preparation. On the other hand, whether functional NMDARs are present in cultured astrocytes is still a matter of debate due to contradictory findings. Kettenman and Schanchner did not find currents in response to NMDA in rCCA [4], whereas in mouse astrocytes Kato et al. [26] found Glu responses that were insensitive to NMDAR inhibitors. Some authors have even claimed unanimous agreement that cultured astrocytes are devoid of functional NMDAR [24, 27]. Nevertheless, to our knowledge at least six studies have reported functional NMDAR in cultured astrocytes from human [28–30] or rat [31, 32, 48]. Two of these studies found an *i*Ca^2+^ rise and/or evoked currents in human astrocytes in response to NMDA, although these effects were not sensitive to APV and only partially sensitive to extracellular Ca^2+^ depletion [28, 30]. In contrast, in human astrocytes, the *i*Ca^2+^ rise and cell death induced by Glu were sensitive to memantine or MK-801, evidence of canonical NMDAR function [29]. Also, in anoxic rat astrocytes, APV-sensitive *i*Ca^2+^ rise was induced by NMDA [48]. Additionally, molecular nuclear translocation was reported in rCCA after NMDA treatment [32]. Recently, in rCCA co-cultured with brain endothelial cells, a canonical NMDAR-mediated *i*Ca^2+^ rise was found and non-canonical NMDAR function was suggested [31]. These apparently contradictory data, together with the belief that non-excitable cells could not have functional NMDARs due to the Mg^2+^ block, argued against functional NMDARs in astrocytes.

In the present work, we detected all NMDAR subunits by IF that exhibited a phenotype characteristic of transmembrane molecules (Figs 1 and 3A and 3C). Intriguingly, we observed a more regionalized distribution for the GluN1 IC domain than for its EC domain. This observation could be related to posttranslational modifications similar to those described in neurons [49, 50]. Previously, labeling for most NMDAR subunits was found in cultured human astrocytes [29]; GluN1 and GluN2A were observed in cultured rat hippocampal astrocytes after anoxia [48], whereas the GluN2 subunit was detected in rCCA co-cultured with brain endothelial cells [31]. In agreement with these observations we found mRNA expression of all NMDAR subunits. Unfortunately, it is impossible to correlate mRNA expression and IF intensities because the Abs we used have different recognition sequence sizes. Previously, mRNA for most NMDAR subunits was detected in cultured human astrocytes; whereas GluN1-2A-2B mRNAs were found in acute isolated mouse astrocytes; whereas rat brain *in situ* hybridization experiments for GluN1 were non-conclusive [24, 29, 51].

Since non-functional NMDAR may result from incorrect GluN1 assembly and transport [15–21], we analyzed its size and localization. We found full-length expression of GluN1 by WB. Interestingly, we observed another conspicuously smaller band, suggesting GluN1 posttranslational modifications (S6 Fig). To our knowledge, this is the first work where GluN1 expression is identified by its M_r_ in astrocytes. A previous study reported efforts using eGFP-tagged astrocytes acutely isolated from mouse brain, but the amounts harvested were insufficient for WB analysis [24]. Thus, rCCA could help to identify NMDAR posttranslational modifications, which are known to regulate its function and proposed to confer its particular electrophysiological properties in astrocytes [5, 7]. We also confirmed the GluN1 plasma membrane localization, suggesting its association with other NMDAR subunits in the ER, because GluN1 also requires its association with other NMDAR subunits that mask its ER retention signals [15, 17, 21].

We next found that NMDA elicited an *i*Ca^2+^ rise in rCCA, although the heterogeneous cell responses suggest that NMDAR activity varies in rCCA. This could be related to: a) different expression levels of NMDAR subunits, and/or b) cell differences in NMDAR regulation and/or assembly. Intriguingly, we observed that *i*Ca^2+^ increases not only in the cytoplasm but apparently also within the nucleus, however more experiments are needed to confirm this possibility (S1 Video). The NMDA effect was specific because two competitive inhibitors blocked this response, although with different efficacy. This could be related to the NMDAR composition, since all NMDAR have GluN1 subunits, whereas different types of GluN2 subunits may be assembled into NMDAR. Furthermore, it is known that GluN2 subunits possess different sensitivities to APV [15]. It is possible that the bimodal distribution of cell responses observed with APV could reflect different NMDAR populations. On the other hand, inhibition by KYNA and NMDA+Gly experiments that did not changed rCCA response (S7 Fig) suggest that the Gly site of GluN1 is activated. This activation could be achieved by D-serine, which is known to be an alternative ligand for the GluN1 Gly site that is secreted in large amounts by astrocytes [52]. Consistent with this idea, we found that GluN1 knock down by siRNA also inhibited the response of rCCA to NMDA. Unexpectedly, we found that a) siC transfection decreased responses to NMDA in comparison with non-transfected cells, and b) the transfection protocol itself resulted in a large population of non-responsive cells. These outcomes could result from remodeling of plasma membrane dynamics or molecular dynamic distortion at plasma membrane as previously reported with Lipofectamine [53], [54, 55].

To our surprise, the NMDA effect on *i*Ca^2+^ rise was mediated by the NMDAR in a non-canonical, flux-independent manner, releasing Ca^2+^ from intracellular pools, resembling the mechanism of a metabotropic receptor. This is supported by *i*Ca^2+^ diffusion observed in time lapses with faster frame rates that show its diffusion from the center to the edges (S1 Video). Non-canonical NMDAR function that is independent of ion-flux has been reported previously by a few groups. One report demonstrated NMDAR down-regulation by GluN1 and GluN2 tyrosine dephosphorylation in an agonist-dependent, ion flux-independent manner [42]. Also, GluN2B exchange by GluN2A at synapses is induced by NMDAR ligand binding but independent of ion-flux [37]. Others demonstrated that NMDAR Gly site activation initiates intracellular signaling, priming the receptor for clathrin-dependent endocytosis [40]. Also, Extracellular Signal-regulated Protein Kinase (ERK) signaling activation by NMDAR and metabotropic Glu receptor 5 co-activation occurs in a Ca^2+^ flux-independent manner [43]. More recently, two groups demonstrated that oligomeric Aβ-induced synaptic depression is due to ion-flux independent, metabotropic-like NMDAR activities [38, 41]. One of these groups also reported that NMDAR mediated LTD depends upon its metabotropic signaling [39]. Thus, despite the fact that NMDAR-mediated Ca^2+^ influx is well established, the studies cited above and the present work support an alternative functional scenario for the NMDAR. More studies are required to fully characterize this scenario, which could explain why electrophysiological experiments ruled out functional NMDARs in cultured astrocytes [4]. This alternative scenario is not entirely unexpected, since non-canonical functions for membrane molecules are becoming more evident [56, 57]. Indeed, to our knowledge, two previous reports hinted that NMDAR mediated Ca^2+^ exit from intracellular pools [30, 31]. Unfortunately, the evidence was not conclusive and did not excluded the possibility that CICR could mediate Ca^2+^ exit from intracellular pools. We did rule out this possibility because MK-801 and extracellular Ca^2+^ depletion did not block or reduced the rCCA response to NMDA.

We also found that tyrosine kinases regulate NMDAR. In this regard, it is well known that the GluN1 and GluN2A-B subunits are subject to tyrosine phosphorylation that regulates NMDAR activity [15]. However, it is possible that other NMDAR subunits also undergo tyrosine phosphorylation, although the functional consequences have not been studied. In addition, other signaling molecules that regulate Ca^2+^ release from the ER could also be implicated. We also contrasted the NMDA effect with that of the well-known gliotransmitter ATP. Although the averaged responses were similar, we found differences in their response distributions that could explained by distinct purinergic and NMDAR expression levels, and/or with their regulatory mechanisms. Interestingly, our approach using ∫(ΔF/F_0_) distribution analysis, may provide additional insight into the cellular and molecular mechanisms and enable a more integrative view of these complex phenomena.

We measured mΔψ considering the role of mitochondria in *i*Ca^2+^ metabolism [33, 34, 58], which has been found to be depleted by NMDAR in neurons [47, 59], using the ratiometric sensor JC-1 [46, 47, 59–61]. The temporal shift between *i*Ca^2+^ rise and mΔψ depletion depends on different cellular mechanisms. First, NMDARs induce the *i*Ca^2+^ rise that reaches maximal levels within seconds; then, mitochondria initiate *i*Ca^2+^ uptake at the expense of mΔψ. Finally, mΔψ takes time to recover, because it requires the activation of mitochondrial electron transport and metabolic pathways. We assume that mΔψ eventually recovers, because the rCCA were alive for weeks after NMDA challenge. Here, it is critical to clarify that our mΔψ measurements comprise populations and not single mitochondria; therefore, temporal scales are different from those described by Keil et al. [46]. On the other hand, we found that NMDA primed cells for a larger mΔψ depolarization after CCCP (Fig 8A); this outcome may be the consequence of the inrplay among cellular mechanisms. We do not know the nature of these putative mechanisms, but they may include mitochondrial ion/proton equilibrium or intracellular signal distortion due to the *i*Ca^2+^ rise. NMDA also elicited heterogeneous mΔψ responses that could be associated with the diversity of *i*Ca^2+^responses, or alternatively, with other cell conditions that may include: NMDAR status, redox conditions, substrate availability, enzyme activity and/or availability and mitochondrial metabolism. These cellular conditions could also help to explain the different number of cells that rise *i*Ca^2+^ of those that depleted mΔψ.

Importantly, the NMDA concentration used in our experiments is within the Glu physiological levels reported within the synaptic cleft [62], where astrocytes extend their protrusions. This concentration (1 mM) was employed by Vissel et al. [42] and previous experiments performed in astrocytes [28–30, 48]. This high NMDA concentration required could be related with NMDAR membrane organization in non-neuronal cells, that contrast with synapses of neurons, where post-synaptic proteins confer specific spatial, structural, trafficking and functional characteristics to NMDAR [63]. Alternatively, biochemical and/or biophysical NMDAR peculiarities in astrocytes or non-neuronal cells may be involved in this. In this regard, posttranslational modifications could regulate its function as suggested elsewhere [5, 7]. On the other hand, NMDAR in acute isolated mouse astrocytes are hetero-trimeric complexes with GluN1, GluN2C-D and GluN3 subunits. This composition is not blocked by Mg^2+^, enabling its activation at physiological resting potentials, but with lower Ca^2+^ permeability than neuronal receptors [23]. Such NMDAR composition is consistent with our observations, but there are other alternatives, since we found expression of GluN2A and GluN2B subunits.

This work employed different approaches to demonstrate that rCCA express non-canonical functional NMDARs with metabotropic-like signaling. This supports that astrocytes are active participants in brain function as proposed by the tripartite synapse hypothesis, in this case through NMDAR as a putative player in *i*Ca^2+^ rise, that occurs in astrocytes in an oscillatory manner, that it may in turn regulate Ca^2+^ dependent secretion [44, 58, 64]. Nevertheless, although NMDAR metabotropic function has been demonstrated in neurons, heterologous systems and brain, more experiments are required to test whether this non-canonical NMDAR function occurs in astrocytes *in vivo*. Moreover, considering previous *in situ* and *in vitro* studies with astrocytes, rCCA seem to exacerbate the metabotropic-like function of the NMDAR, a fact that could represent an advantage to study this NMDAR function. These findings also open the question about how NMDAR function, their mΔψ depolarization and their metabolic outcomes in astrocytes are involved in neuronal death, survival and communication, considering the close interaction between these two cell types in health and disease in the CNS.

## Supporting Information

S1 Fig rCCA characterization. (A) GFAP labeling of rCCA. (B) High magnification of representative cells showing GFAP phenotype. (C) Glutamine synthase (Gln Syn) labeling of rCCA. (D) High magnification of representative cell showing Gln Syn phenotye. (E) Texas red hydrazide (Tex Red H) (Sulforhodamine 101 fixable analogue) vital labeling of rCCA. (F) MAP2 labeling of rCCA; as expected no cells were positive since neurons do not proliferate nor do they survive trypsinization. All images are representative of each staining. For GFAP, Gln Syn and Tex Red H <95% of cells were stained. Bar = 50μm for A, C, E and F; for B and D = 10μm.
(TIF)

S2 Fig q-PCR positive controls for NMDAR subunit probes. Amplification curves for the indicated gene products with cDNA obtained from rat brain. One representative experiment for each probe is shown.
(TIF)

S3 Fig *i*Ca2+ response in rCCA treated with 100 μM NMDA. rCCA were labeled with Fluo-4 AM, recorded as described in the materials and methods section and perfused with 100 μM NMDA. As observed, this treatment did not modify the averaged *i*Ca^2+^ response. One representative experiment is shown.
(TIF)

S4 Fig GluN1 Knock Down efficiency with different siRNA concentration. rCCA seeded into 24 well plates were transfected with 2 μl/ml Lipofectamine 2000 and the indicated nM concentration of siRNA (siC or siGrin1). 24 h after cells were fixed, stained against extracellular NMDAR subunit GluN1, photographed (40X; N.A. 1.35) and analysed. Data represent average fluorescence per cell ± s.e.m. from 35–60 cells evaluated with background subtracted obtained from control cells without primary Ab. Higher amounts of Lipofectamine 2000 or siRNA caused substantial cell death and detachment.
(TIF)

S5 Fig mΔψ response in rCCA treated with 100 μM NMDA. rCCA were labeled with JC-1, recorded as described in the materials and methods section and incubated with 100 μM NMDA. As observed, this treatment did not change mΔψ. One representative experiment is shown.
(TIF)

S6 Fig Western Blot (WB) of rCCA lysates. rCCa lysates were prepared and WB was performed with an Ab against the EC domain of GluN1 subunit as described in the materials and methods section. As observed, a conspicuous band was detected below the full-length GluN1 (115 kDa). One representative experiment is shown.
(TIF)

S7 Fig *i*Ca2+ response in rCCA treated with 1 mM NMDA and 100 μM Gly. rCCA were labelled with Fluo-4 AM, recorded as described in the materials and methods section and then perfused with 1mM NMDA+100 μM Glycine (Gly). As observed, this treatment increased the averaged *i*Ca^2+^ response with a time course similar that of NMDA alone (see text). One representative experiment is shown.
(TIF)

S8 Fig Representative images from iCa2+ recordings in the different experimental conditions. For each experimental condition discussed in the text (rows) a representative cell was chosen and three frames were extracted from the recording representing basal (left column), peak (middle column) and end (right column) conditions.
(TIF)

S1 Video (MPEG)
