# Correction: A Metabotropic-Like Flux-Independent NMDA Receptor Regulates Ca^2+^ Exit from Endoplasmic Reticulum and Mitochondrial Membrane Potential in Cultured Astrocytes

**DOI:** 10.1371/journal.pone.0202819

**Published:** 2018-08-20

**Authors:** Pavel Montes de Oca Balderas, Penélope Aguilera

A sentence is missing from the “Intracellular calcium (*i*Ca^2+^) measurements” subsection of the Materials and Methods. The subsection should read: *i*Ca^2+^ determinations were performed as described previously [36] with modifications. rCCA were loaded for 45 min with 1 μM Fluo-4-AM, 0.02% pluronic acid in HBSS with 1.2 mM CaCl_2_, 400 μM MgSO_4_ and 440 μM MgCl_2_ (referred to as vehicle). HBSS with 1 mM NMDA reaches a pH near 6. Coverslips were transferred to a stage chamber and fluorescence emission was recorded with a 10X 0.4 N.A. objective with the microscope set up described above. Fluorescence was recorded with a 485/30 bandpass excitation filter, a 505 longpass dichroic mirror and a 510/50 bandpass emission filter. Time-lapse recordings were acquired at 20 frames per min (f/m). After 120 sec recording basal fluorescence with vehicle perfusion at 400 μl/min with or without inhibitors, NMDA, ATP or vehicle was perfused and recording continued for another 230 sec. Before the experiment, cells were treated 15–45 min with inhibitors that were constantly perfused during the recording. Time–lapses were analyzed with Cell Sens Dimension software. Background noise was subtracted and oval regions of interest (ROI) were drawn around each cell soma. Labeling phenotypes showing bright cytoplasmic puncta were discarded. The mean fluorescence intensity (F) was obtained for each ROI’s and *i*Ca^2+^ responses were evaluated by calculating the relative fluorescence change (ΔF/F_0_) with the formula ΔF/F_0_ = (F*t*_*x*_-F_0_)/F_0_, where F*t*_*x*_ is F at time *x* and F_0_ is the average fluorescence during the first 50 sec. Data are presented as average response traces with cells obtained from at least three different cultures. The integrated relative fluorescence change ∫(ΔF/F_0_) for each cell was calculated as the sum of normalized ΔF/F_0_ values between 0 and 350 sec. ∫(ΔF/F_0_) values for the cell populations tested are presented as distribution histograms for each experiment. The number of cells (*n*), experiments and numerical descriptors for averaged responses and distribution histograms are found in Table 1. For experiments in Ca^2+^-free conditions cells were pre-incubated for at least 15 min and up to 45 min before recording.
