# Supplementary figures and images for: A Metabotropic-Like Flux-Independent NMDA Receptor Regulates Ca2+ Exit from Endoplasmic Reticulum and Mitochondrial Membrane Potential in Cultured Astrocytes
Source: PLoS One. 2015 May 8;10(5):e0126314. doi: 10.1371/journal.pone.0126314 (PMC4425671; doi:10.1371/journal.pone.0126314)

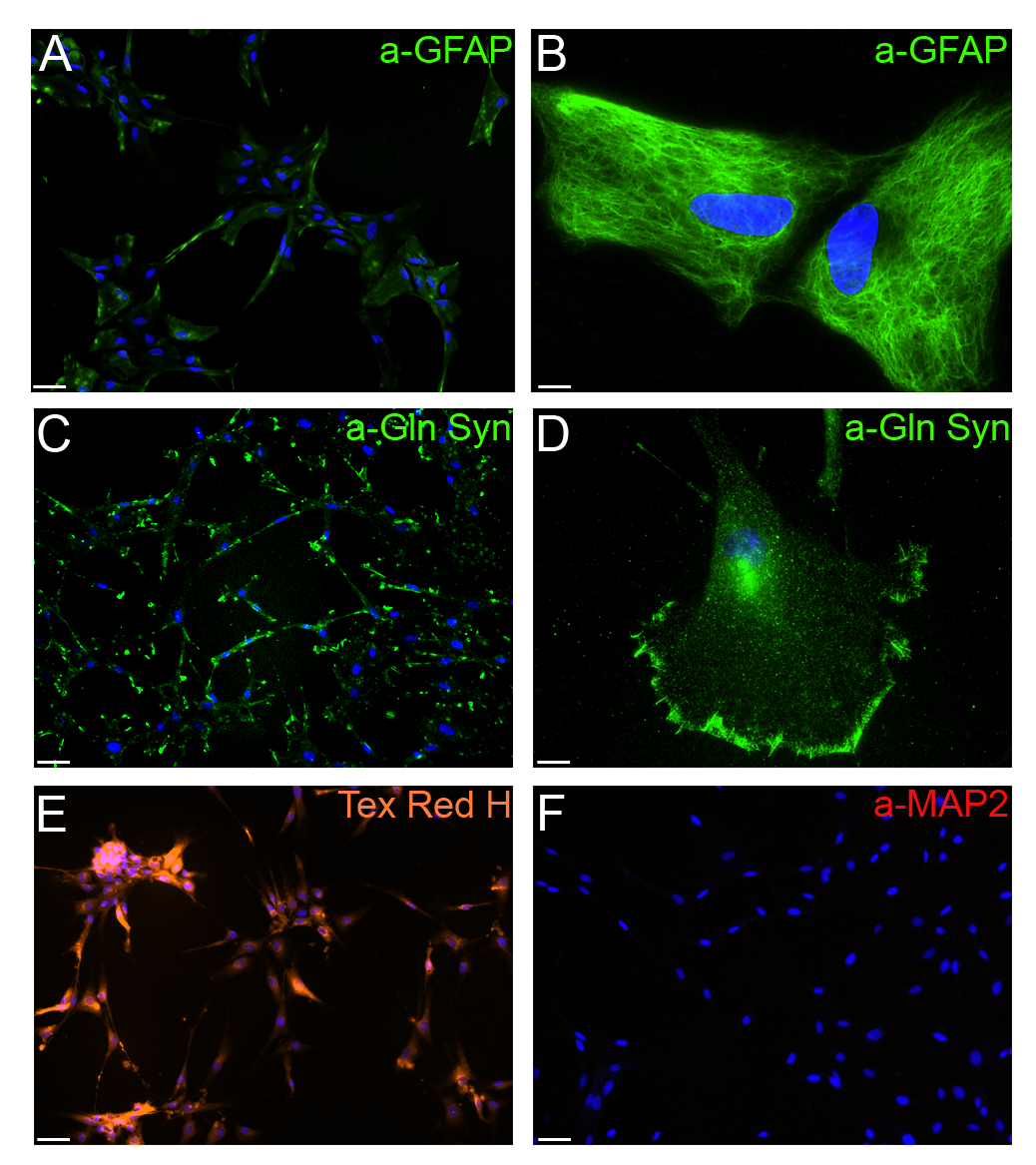

Supplement: S1 Fig — (A) GFAP labeling of rCCA. (B) High magnification of representative cells showing GFAP phenotype. (C) Glutamine synthase (Gln Syn) labeling of rCCA. (D) High magnification of representative cell showing Gln Syn phenotye. (E) Texas red hydrazide (Tex Red H) (Sulforhodamine 101 fixable analogue) vital labeling of rCCA. (F) MAP2 labeling of rCCA; as expected no cells were positive since neurons do not proliferate nor do they survive trypsinization. All images are representative of each staining. For GFAP, Gln Syn and Tex Red H <95% of cells were stained. Bar = 50μm for A, C, E and F; for B and D = 10μm. (TIF) [file pone.0126314.s001.tif]

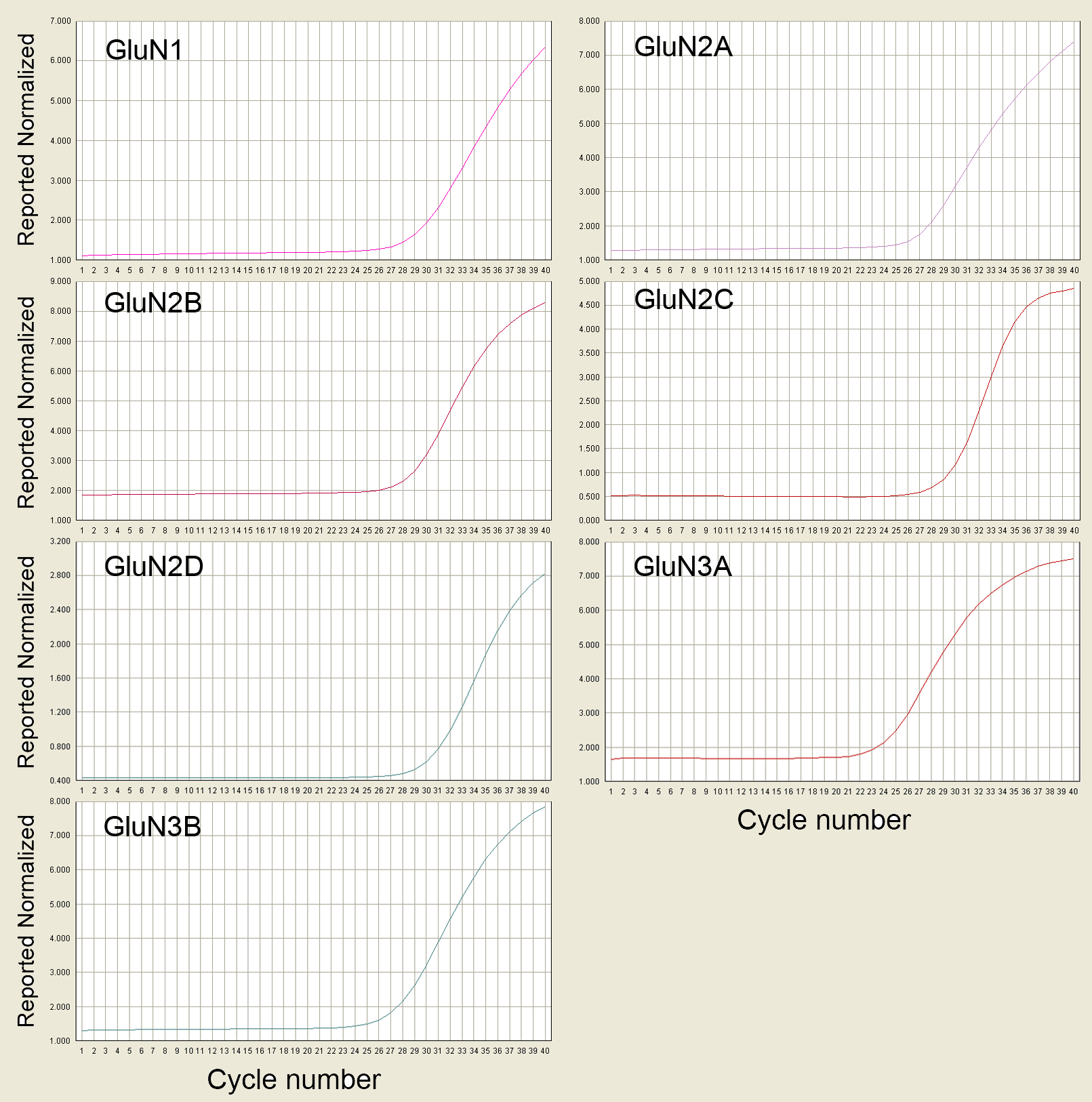

Supplement: S2 Fig — Amplification curves for the indicated gene products with cDNA obtained from rat brain. One representative experiment for each probe is shown. (TIF) [file pone.0126314.s002.tif]

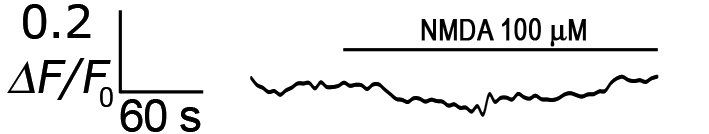

Supplement: S3 Fig — rCCA were labeled with Fluo-4 AM, recorded as described in the materials and methods section and perfused with 100 μM NMDA. As observed, this treatment did not modify the averaged iCa2+ response. One representative experiment is shown. (TIF) [file pone.0126314.s003.tif]

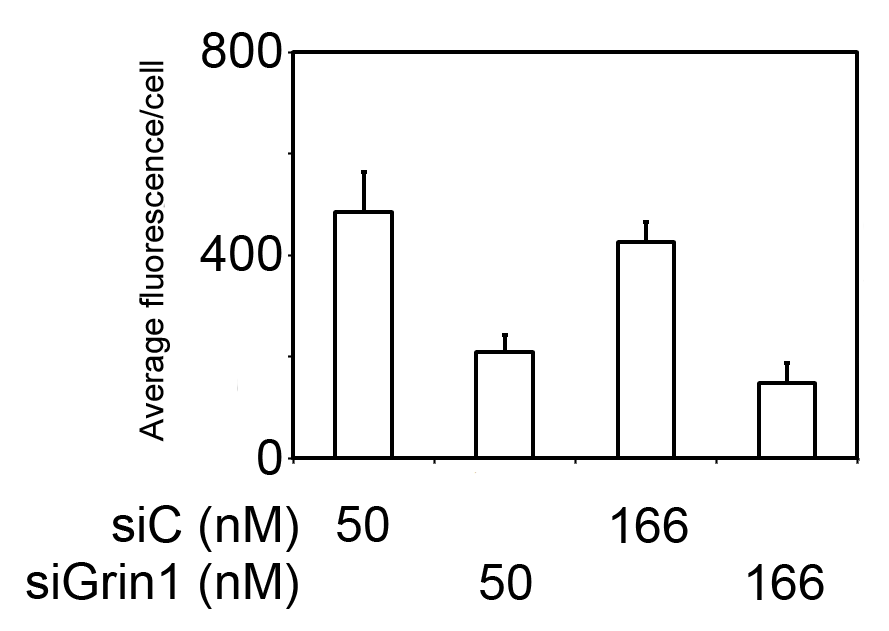

Supplement: S4 Fig — rCCA seeded into 24 well plates were transfected with 2 μl/ml Lipofectamine 2000 and the indicated nM concentration of siRNA (siC or siGrin1). 24 h after cells were fixed, stained against extracellular NMDAR subunit GluN1, photographed (40X; N.A. 1.35) and analysed. Data represent average fluorescence per cell ± s.e.m. from 35–60 cells evaluated with background subtracted obtained from control cells without primary Ab. Higher amounts of Lipofectamine 2000 or siRNA caused substantial cell death and detachment. (TIF) [file pone.0126314.s004.tif]

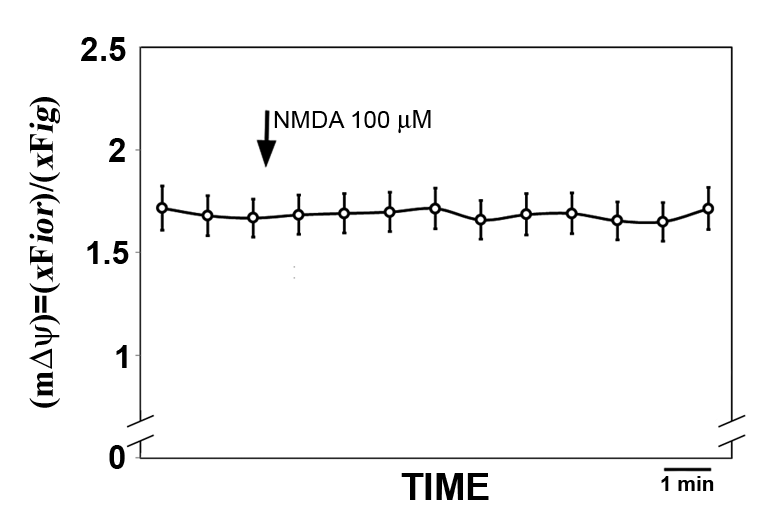

Supplement: S5 Fig — rCCA were labeled with JC-1, recorded as described in the materials and methods section and incubated with 100 μM NMDA. As observed, this treatment did not change mΔψ. One representative experiment is shown. (TIF) [file pone.0126314.s005.tif]

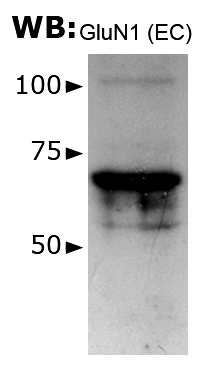

Supplement: S6 Fig — rCCa lysates were prepared and WB was performed with an Ab against the EC domain of GluN1 subunit as described in the materials and methods section. As observed, a conspicuous band was detected below the full-length GluN1 (115 kDa). One representative experiment is shown. (TIF) [file pone.0126314.s006.tif]

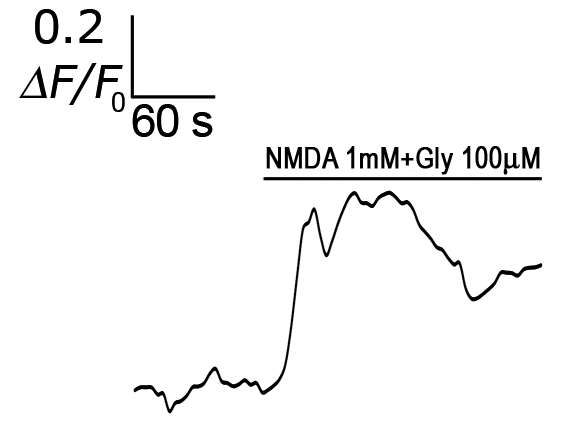

Supplement: S7 Fig — rCCA were labelled with Fluo-4 AM, recorded as described in the materials and methods section and then perfused with 1mM NMDA+100 μM Glycine (Gly). As observed, this treatment increased the averaged iCa2+ response with a time course similar that of NMDA alone (see text). One representative experiment is shown. (TIF) [file pone.0126314.s007.tif]

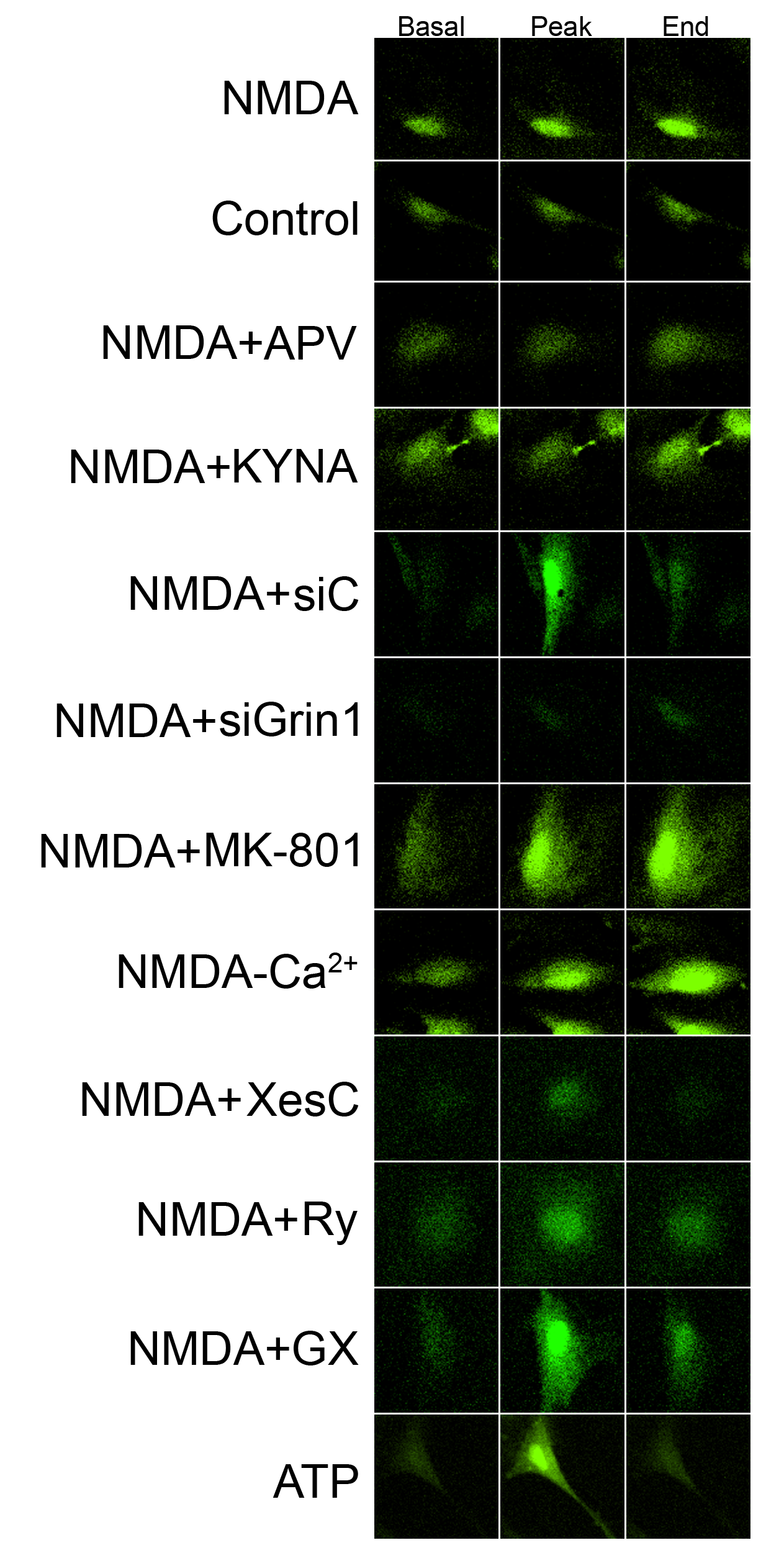

Supplement: S8 Fig — For each experimental condition discussed in the text (rows) a representative cell was chosen and three frames were extracted from the recording representing basal (left column), peak (middle column) and end (right column) conditions. (TIF) [file pone.0126314.s008.tif]
